# Evaluation of clinical, etiological and antimicrobial resistance profile of pediatric urinary tract infections in a secondary health care centre

**DOI:** 10.4314/ahs.v21i2.10

**Published:** 2021-06

**Authors:** Gökce Celep, Hüseyin Burak Özçelik

**Affiliations:** 1 Amasya University, Faculty of Medicine, Department of Pediatrics, Amasya, Turkey; 2 Amasya University, Sabuncuoğlu Şerefeddin Education and Research Hospital, Microbiology Laboratuary, Amasya, Turkey

**Keywords:** Urinary tract infections, antibiotics, susceptibility

## Abstract

**Background:**

Urinary tract infections are common during childhood. The etiologic agents and empirical antibiotics may vary due to age and geographic area.

**Objectives:**

This study was designed to investigate the urinary tract infection pathogens, their antibiotic resistance profile and risk factors in a sample of well-child population.

**Materials and Methods:**

This retrospective study was conducted in the pediatric clinics of a secondary health-care centre in a one-year period. The source of data was hospital and laboratory records. Toilet trained children and adolescents aged between 5–17 years old with positive urine culture were enrolled into the study. Microbiological studies were conducted according to international guidelines.

**Results:**

During the study 3640 urine samples were analyzed and 342(9.4%) had significant growth. Gram negative enterobacteria were the most common infectious agents. Antibiotic susceptibility tests showed low cephalosporine resistance unless ESBL was positive. Multi drug resistance was remarkable. Extended beta lactamase resistance rate was 17%. Previous history of antibiotic use before the present administration was the only significant risk factor for ESBL positivity.

**Conclusion:**

Treating urinary tract infections may become an emerging problem soon. Unless there are risk factors, cephalosporines are good options, but if so nitrofurantoin or carbapanems should be preferred for treatment in this population.

## Introduction

Urinary tract infections (UTI) are common febrile diseases during childhood. In fever of unknown origin, UTI must be considered as a differential diagnosis.^1^ The prevalence of pediatric UTI was reported as 2–20% worldwide^1^,^2^. The infection has no specific signs and symptoms that is why clinical suspicion and laboratory tests are important.^3^ After the first episode, recurrent infections can occur within 6–12 months^4^. The infection may be community acquired or as a complication related to hospitalization by increasing health care costs and morbidity rate^5^. Timely diagnosis and effective antibiotic therapy can prevent the complications of UTI. Infection itself may cause morbidity and mortality due to pyelonephritis and septicaemia. Also, renal scarring, chronic renal failure, hypertension may be the long term complications^6^–^8^. The etiologic agents are determined by culture studies, but empirical antibiotics according to age and geographic data are often prescribed before the culture results and antibiogram reports^9^. However increasing antibiotic resistance of urinary system pathogens is a global problem. Inappropriate and widespread use of antibiotics has led to multidrug resistant (MDR) pathoens^10^. The right choice and effective dose are also important to prevent nephrotoxicity^11^. The antimicrobial susceptibility of the microorganisms and risk factors causing multi drug resistance may vary regionally^9^,^12^. Bacteria gain resistance to beta-lactam antibiotics (synthetic penicillins, cephalosporins, aztreonam) and this makes the therapy process complicated with poor outcome and/or application of expensive, broad spectrum antibiotics such as carbapenems^13^,^14^. Rising carbapenem resistance is another problem^15^. The rise in extendedspectrum -beta lactamase (ESBL) producing bacteria in UTI is a therapeutic problem in children since the treatment options are limited and not suitable for outpatient settings^16^. Infancy, uroprophylaxis, recent antibiotic therapy, recurrent UTI, male gender have been reported as risk factors for UTI with ESBL-producing organisms^17^. ESBL producing organisms present an ever-growing burden not only for hospitalized patients, but for community settings as well^18^,^19^.

The infectious agents and their antibiotic resistance patterns may vary between populations and regions. This study was designed to investigate the urinary tract infection pathogens, their antibiotic resistance profile and risk factors related with these parametres in a sample of previously healthy pediatric population who were admitted to the pediatric clinics of a secondary health care center. We aimed to emphasize risk factors for ESBL (+) uropathogens and recommend therapeutic choices available for children suffering from UTI.

## Material and Methods

### Study design and data collection

This retrospective study was conducted between 1^st^ April, 2016 and 31^st^ March, 2017 in the pediatric clinics of a secondary health- care centre in the middle-northern Turkey. The source of data was hospital and laboratory records. All toilet trained patients aged between 5 and 17 years old with positive urine culture, either admitted to pediatric outpatient clinics or hospitalized, were enrolled into the study. Children having no toilet training were excluded as urine bag samples or collecting urine via catheterization was not suitable for standardization. Patients with risk factors for recurrent UTI such as urogenital anomalies or neuromuscular problems related with non-optimal urine drainage were also excluded. Only one positive culture per patient was included in the study and repeated cultures from the same patient at the same time were excluded from the analysis. All urine specimens were obtained by midstream clean-catch method in toilet-trained children.^19^ Bag urine samples were not taken in consideration. Age, gender, hospital admission within 3 months, hospitalization status at the time of positive sample, season, the microorganisms in the urine culture, their antibiotic susceptibility profile, ESBL status, prescribed antimicrobial drugs 3 months before the positive urine specimen were noted through electronic hospital record system (Sisoft HBYS®). “Laboratory UTI” was defined as “positive urine dipstick for nitrites and/or leukocyte count >5/ HPF. “Positive urine culture” was defined as “monomicrobial culture ≥100,000 colony-forming units [CFU]/mL for midstream and catheter urine”. UTIs were classified as community acquired or healthcare associated due to patients' history. Community-acquired UTI (CA-UTI) was defined as a UTI episode in which, at the time the index urine sample was submitted, the patient was not hospitalized and had not been previously hospitalized during the preceding 30 days. Healthcare associated infection (HAI) was defined as “infections that patients acquire during the course of receiving healthcare treatment” 5,14.

### Microbiological procedures

The sterile mid-stream urine specimens were planted on 5% blood agar and Eosin Methylene-blue Lactose Sucrose Agar (EMB) by semi-quantitative planting method with calibrated loop (0.001 ml). Planted plates were incubated aerobically at 37°C for 18–24 hours. Samples having significant growth (that is ≥10^5^ colony forming units (CFU/ml)) were processed for further identification and susceptibility testing via VITEK 2 Compact (Biomerieux-France) system according to the manufacturer's instructions. Antimicrobial susceptibilities and ESBL production were determined due to the European Committee on Antimicrobial Susceptibility Testing guidelines^20^. The microorganisms reported as ESBL (+) by VITEK went through confirmation tests by double disc synergy method that tests the susceptibility of the strain against amoxycillin and amoxycillin /clavulanic acid. The strain that showed increased inhibition zone with the combination disc on Mueller- Hinton agar plate was considered to have ESBL. E.coli ATCC 25922 was used as control test.

### Statistical analyses

The analyses were performed using SPSS version 15 (SPSS, Inc., Chicago, IL, USA). The data were presented as frequencies, medians and minimum-maximum, range or mean ± SD by descriptive statistics, when indicated. Cross- tabs with chi-square test (χ2) and z-test were used to identify statistically significant differences between groups at 95% confidence. Probability factor (p) less than 0.05 was regarded as statistically significant.

### Ethics statement

The Ethical Committee of Hitit University approved this study (approval number: 2019-103)

## Results

During the study period (1^st^ April, 2016-31^st^ March, 2017), 8381 urine samples were accepted to the microbiology laboratory for culture tests and 3640 samples were suitable for this study. The samples belonging to children under five years old and having no toilet training (n= 4710) or patients with risk factors for recurrent UTI such as urogenital anomalies or neuromuscular problems related with non-optimal urine drainage (n=31) were excluded. Among 3640 UTI suspected samples, 342 (9.4%) had significant growth. Of the 342 isolates 33 (9.6%) belonged to males and 309 (90.4%) to females and the mean age of the study group was 9.04±3.46 years old. Eighty-one (23.7%) of the samples were obtained in winter, 160 in autumn and spring equally and the rest (n=101; 29.5%) in summer. Only 17 (4.9%) samples belonged to hospitalized patients (HAI) and the rest belonged to the patients of outpatient clinics (community acquired infections). Most of the specimens were from the pediatric emergency service (n=189; %55.3). Abdominal pain (n=89; 26.1%), dysuria (n= 45; 13.2%), nausea and vomiting (n= 12.9%) were the most common symptoms during administration (Table 1).

**Table 1:** Clinical features of the study population (page 7)

Age	9.04±3.46 (5–17) years	

**Gender**		

Girls	2538	69.7%
Boys	1102	30.3%

**Season**		

Autumn	80	23.4%
Winter	81	23.7%
Spring	80	23.4%
Summer	101	29.5%

**Service**		

Pediatric Emergency Service	189	55.3%
Pediatrics	105	30.7%
Pediatric Surgery	48	14%

**Patient setting**		

Outpatient clinics	325	95%
Hospitalized	17	5%

**Reason for administration**		

Abdominal pain	89	26.1%
Dysuria	45	13.2%
Nausea ± vomiting	24	7.0%
Fever	20	5.8%
Enuresis	7	2.0%
Anorexia	2	0.6%

**Dipstick test**		

Compatible with UTI	212	62 %
Non- compatible with UTI	54	15.8%

**Patient history**		

Hospital administration before the index positive urine culture	1.89±2.29 (median:1)	
Hospitalization before the index positive urine culture	10	
Antibiotics (box per patient)	0.77±1.04 (median: 1)	

Among the study group, 218 patients were admitted to the hospital at least once (range: 0–13) and 10 were hospitalized three months before the positive urine culture result. According to the accessed records 79 had respiratory tract infections, 53 had UTI and 26 acute diarrhea and vomiting . Approximately half of the patients (n=156; 45.6%) had received several antibiotics at different times within the preceding three months. Amoxicillin clavulanic acid (n= 71; 29.1%), cefuroxime (n= 44; 18%), cefixime (n= 27; 11%) were the most frequent prescribed antimicrobials (Table 2). Seventeen patients were hospitalized during sample obtaining, diagnosed as HAI and two of them had urine catheter in intensive care unit.

**Table 2:** The prescribed antibiotics before the index positive urine culture (page 7)

Antimicrobial Agents	n	%
Amoxicillin clavulanic acid	71	29.1 %
Cefuroxime	44	18 %
Cefixime	27	11 %
Co-trimaxazole	15	6.2 %
Ceftriaxone	13	5.3 %
Claritromycine	12	4.9 %
Cefdinir	12	4.9 %
Nitrofurantoin	10	4.1 %
Oseltamivir	5	2 %
Metronidazole	7	2.9 %
Sulbactam-Ampicillin	5	2 %
Gentamicin	4	1.6 %
Phosphomycine	4	1.6 %
Penicillin	3	1.2 %
Azithromycin	3	1.2 %
Amikacin	1	0.4 %
Others	8	3.3 %

(Flucanazole, spiramycine, cefpodoxime, ciprofloxacin, cefazoline, cefalexine)

Dipstick tests were performed for 266 patients and 79.7% (n= 212) of the results were compatible with laboratory UTI. Gram negative bacilli were the most common infectious agents and *E.coli* was leading (n=268; 78.4%). The rate of Gram positive agents was 6.4% (n=22) (Table 3). Antibiotic susceptibility tests showed low cephalosporine resistance unless ESBL is positive; however, ampicillin and co-trimoxazole seem to be inappropriate options for empiric treatment in our population. The rate of oxacillin resistance in Gram positive agents was 50% (n=11) and there was no vancomycin resistance in enterococci (Table 4).

**Table 3:** Distribution of infectious agents (page 8)

	n	%
Escherichia coli	268	78.3
Gram (+) cocci	22	6.5
*Klebsiella* strains (spp)	19	5.6
*Enterococcus* spp	12	3.6
Proteus mirabilis	8	2.4
*Pseudomonas aeruginosa*	4	1.2
*Enterobacter* spp	4	1.2
Others (*Acinetobacter* spp, *Citrobacter* spp	5	1.3
**TOTAL**	**342**	**100**

**Table 4:** Infectious agents and their antibigram results

Sensitivity(S)/Resistance % S	Ampicillin	Amoxicillin Clavulanic Acid	Piperacillin Tazobactam	Cefuroxime	Cefoxitine	Cefixime	Ceftazidime	Ceftriaxone	Ertapenem	Imipenem	Meropenem	Amikacin	Gentamicin	Ciprofloxacine	Phosphomycine	Nitrofurantoin	Co-trimaxasozole
E.coli n=268	118/16 47.0% (251)	149/101 59.6% (250)	221/26 98.4% (247)	200/51 79.7% (251)	231/20 92.0% (251)	195/ 11 83.7% (233)	207/41 83.5% (248)	211/37 85.1% (248)	248/2 99.2% (250)	231/1 99.5% (232)	246/3 98.8% (249)	245/6 97.6% (251)	234/17 93.2% (251)	214/37 85.3% (251)	230/3 98.7% (233)	231/2 99.1% (233)	184/66 73.6% (250)
Klebsiella spp n=19	1/18 5.3% (19)	10/9 52.6% (19)	12/7 63.1% (19)	15/4 78.9% (19)	17/2 89.5% (19)	13/3 81.3% (16)	15/4 78.9% (19)	13/6 68.4% (19)	17/2 89.5% (19)	15/1 93.7% (16)	19/0 100% (19)	19/0 100% (19)	18/1 94.7% (19)	17/2 89.5% (19)	13/3 81.2% (16)	15/1 93.7% (16)	16/3 84.2% (19)
Proteus spp n=8	4/4 50% (8)	6/2 75% (8)	8/0 100% (8)	6/2 75% (8)	8/0 100% (8)	8/0 100% (8)	6/2 75% (8)	5/3 62.5% (8)	7/1 87.5% (8)	8/0 25% (8)	8/0 100% (8)	8/0 100% (8)	8/0 100% (8)	8/0 100% (8)	8/0 100% (8)	5/3 62.5% (8)	5/3 62.5% (8)
Enterococcus spp n=12	7/3 (10)													8/2 80% (10)			7/3 70% (10)
Pseudomonas aerogineosa n=4			3/0 100% (3)				3/0 100% (3)			1/1 50% (2)	2/1 66.6% (3)	2/1 66.6% (3)	2/1 66.6% (3)	2/1 66.6% (3)			
Staphylococcus spp n=18													13/1 92.8% (14)	13/1 92.8% (14)	6/8 42.8% (14)		14/0 100% (14)

ESBL positivity was detected in 58 (17%) of the samples and these bacteria were susceptible to carbapenems, amikacin, phosphomycin, nitrofurantoin and piperacillin tazobactam (Table 5). Thirty-five (60%) of the ESBL positive patients had previous antibiotic therapy history before present administration and antibiotic use was statistically significant for ESBL positivity (p= 0.01). ESBL was more frequent in females, however there was no statistical significance between genders (p= 0.15). Most of the ESBL (+) patients (n=48; 82.8%) were under 12 years old, the rate of positivity decreased with age, but this was not statistically significant, either (p=0.44). Also, UTI history within the three months had no significant effect on ESBL positivity (p=0.14). Only one ESBL (+) patient had HAI, all others were evaluated as CA-UTI. After diagnosis, 18 patients were hospitalized for UTI treatment. Among 58 ESBL (+) patients only six were hospitalized and 4 were treated with carbapenems and 2 with amikacin. The rest of the patients were treated with nitrofurantoin (n=34), intramuscular amikacin (n= 5) and gentamicin (n=9) in the outpatient settings. ESBL positivity was not a criterion for hospitalization (p=0.11)

**Table 5:** Antibiogram susceptibility of the ESBL(+) agents

Sensitivity (S)/Resistance (R)% S	Ampicillin	Amoxicillin Clavulanic Acid	Piperacillin Tazobactam	Cefuroxime	Cefoxitine	Cefixime	Ceftazidime	Ceftriaxone	Ertapenem	Imipenem	Meropenem	Amikacin	Gentamicin	Ciprofloxacine	Phosphomycine	Nitrofurantoin	Cotrimaxasozole
*E.coli* n=50	2/44 4.3% (46)	12/34 26% (46)	39/7 84.7 (46)	7/39 15.2% (46)	32/14 69.5% (46)	9/35 20.4% (44)	6/38 13.6% (44)	13/33 28.2% (46)	45/1 97.8% (46)	43/1 97.7% (44)	45/1 97.8% (46)	42/4 91.3% (46)	38/8 82.6% (46)	27/19 58.7% (46)	43/1 97.7% (44)	43/1 97.7% (44)	23/23 50% (46)
*Klebsiella* spp n=4	0/4 0 (4)	0/4 0 (4)	1/3 25% (4)	1/3 25% (4)	4/0 100% (4)	1/2 66.6% (3)	1/3 25% (4)	0/4 0 (4)	4/0 100% (4)	3/0 100% (3)	4/0 100% (4)	4/0 100% (4)	3/1 75% (4)	3/1 75% (4)	3/0 100% (3)	2/1 66.6% (3)	2/2 50% (4)
Enterobacter cloacea n=3	0/3 0 (3)	0/3 0 (3)	0/2 0 (2)	0/3 0 (3)	0/3 0 (3)	0/2 0 (2)	0/3 0 (3)	0/3 0 (3)	3/0 100% (3)	2/0 100% (2)	3/0 100% (3)	3/0 100% (3)	3/0 100% (3)	3/0 100% (3)	1/1 50% (2)	2/0 100% (2)	3/0 100% (3)
Citrobacter freundii n=1	0/1 0 (1)	0/1 0 (1)	1/0 100% (1)		0/1 0 (1)	1/0 100% (1)	0/1 0 (1)	0/1 0 (1)	1/0 100% (1)		1/0 100% (1)	1/0 100% (1)	1/0 100% (1)	0/1 0 (1)			1/0 100% (1)

## Discussion

Urinary tract infections are common problems in the pediatric population. The diagnosis and treatment may be challenging sometimes, but morbidity and mortality can be inevitable due to short and long- time outcomes, unless it is well treated^6^–^8^. Empirical treatment strategies are important because studies to detect the infectious agents and their antimicrobial susceptibility take time. The agents and their antibiotic resistance profile may vary between different areas, age groups, clinical status and may alter within time^21^,^22^. In this retrospectively designed study we focused on culture positive UTI, related risk factors and treatment choices for this common problem.

During a one year period 3640 urine specimens from toilet trained children over 5 years old were evaluated and 342 had significant growth; the rate of culture positivity was 9.6%. UTI prevalence is high throughout the world, it was reported as 16% from Nepal, similar to South- East Asia, but lower in the USA and Iran 9%, 7.87% respectively^9^, ^23^, ^24^. The rates of UTI may be different in different areas. According to the clinical and sociodemographic features, results may vary. Our study group consisted of children who were able to give clean catch specimen and with no risk factors related to recurrent infection. This may be the reason of lower UTI prevalence in this study group. The ratio between females and males in the study population was 2.3 and 9.3 within the culture positive group. The high rate in girls is thought to be related with the anatomic structure. Low rate in boys may be the result of circumcision as it prevents the entrance of microorganisms to the urinary tract from the prepuce^25^. In Turkey most of the boys have circumcision before age 6 years due to regional rules and tradition. The rate of UTI may vary between genders due to age^9^, however age and gender had no statistical significance in our study although infection rate decreased with age and it was high in females.

In children, UTI presents with non-specific symptoms; especially in young children as they cannot express themselves in words. Fever, irritability, anorexia, abdominal pain, nausea, vomiting, urinary symptoms such as dysuria, polyuria, vesical tenesmus, urgency, incontinence are the common problems at admission.^9^, ^26^. Fever, poor feeding, irritability were the most frequent symptoms in younger children as reported in many studies. The members of this study group were at the age that they can express themselves and abdominal pain and/or dysuria were the most common reasons for admission. The symptoms directed the practitioners to urine tests and positivity/ negativity ratio of dipstick tests were compatible with positiveculture results (212/44).

The most common detected microorganism causing UTI was *E.coli* in our study group as it is in many reports^9^,^12^. *E.coli* originate from the faecal flora, spread to the urinary tract and become an infectious agent irrespective to age, gender or season26.Gram negative bacilli are responsible for majority of UTI, but recently Gram positive microorganisms have been also reported as it is in our study^9^.

Multi drug resistance (MDR) to antimicrobial agents is a global public health problem. One of the mechanisms of resistance is to produce ESBL which makes the treatment unresponsive to first choice antibiotics such as cephalosporins for UTI. This increases the morbidity of infection and health care costs via long hospital stay or maltreatment complications. The rate of third generation cephalosporin susceptibility was over 85% in ESBL negative agents. In this study, the rate of ESBL positivity was 17%, lower than many countries. The susceptibility profile consisted of carbapenems (98–100%), phosphmycine (98–100%), aminoglycosides (91–100%); compatible with the literature. Nitrofurantoin susceptibility was approximately 98% to *E.coli*, but 66.6% to Klebsiella spp. Carbapenems have the lowest resistance rate for ESBL (+) agents among other antibiotics^27^. However they are expensive and applied by infusion which makes hospitalization necessary; they are good choices for severe infections or when resistance exists to other antimicrobials^28^. Also they are under cover of the social insurance institution when the patient is hospitalized and one cannot obtain these kind of drugs without formal procedures in our country. In this study 4 patients were treated with meropenem at the hospital. Faropenem and Tebipenem are oral forms of carbapenems, but they are not in the market of our country and experience in the pediatric population is limited^29^.

The role of narrow spectrum antibiotics is popular and nitrofurantoin is a good option with low resistance rate, for *E.coli*. It must be administered four times a day and this makes the patient compliance difficult, but it is applied orally^30^. When UTI is uncomplicated nitrofurantoin seems to be a suitable drug for our population with resistance rate as 0.9% for *E.coli*. The rate of phosphomycine susceptibility in ESBL (+) agents was approximately 100% in this study group. Phosphomycine is applied once orally, this provides excellent patient compliance, however experience is limited in children30. Amikacin is one of the most popular agents in pediatric UTI treatment practice with its long experience history. It was a good choice against Klebsiella spp in this group with a susceptibility rate of 100%. A recent study recommends once-daily intramuscular amikacin as an alternative option for outpatient treatment of community-acquired lower UTIs, but the patients must be monitored for nephrotoxicity and ototoxicity^14^. Co-trimoxazole is another choice in USA, but in Turkey resistance rate is high^31^,^32^. An antibiotic should not be recommended when the resistance rate is over 20% for empirical treatment^31^. The rate of resistance was approximately 25% in our study group, revealing that it is not a suitable choice for our population. Quinolones are other alternative options for the adult population, but they are not recommended for children because of osteoarticular side effects and increasing resistance rate unless UTI is complicated^31^, ^33^. Piperacillin tazobactam may be considered as an alternative option in our population against *E.coli*, varying from other reports^9^.

In this study group 18 patients were hospitalized for UTI treatment. Six of them were infected by ESBL microorganisms. This showed that the clinical status (poor feeding, vomiting, drug intolerance, and lassitude) of the patient is the significant parameter that effects the clinician's decision about hospitalization, not the microorganisms. Among 58 ESBL (+) patients just six were hospitalized and 4 were treated with carbapenems and 2 with amikacin. The rest of the patients were treated with nitrofurantoin (n=34), intramuscular amikacin (n= 5) and gentamicin (n=9) in the outpatient settings.

Risk factors for ESBL positivity were defined as “having urogenital anomalies or neuromuscular problems related with non-optimal urine drainage, clean intermittent catheterization necessity, <1 year-old age, having high UTI recurrence rate, long duration of prophylaxis, use of cephalosporin for prophylaxis, hospitalization within previous 3 months” by Kızılca et al^17^. Our study group consisted of >5 years old children having no risk factors defined by Kızılca et al., but these must be always in consideration while managing UTI. The only risk factor we could define was antibiotic intake within the preceding three months before the culture test. Gender, age, season, UTI history of previous UTI had no statistically significant effect on ESBL positivity.

## Conclusion

We recommend that preventing UTI is easier than treating the infection. Keeping the perineum clean, not using wet wipes for cleaning, wiping without contaminating anal region, keeping away ointments containing steroids for perianal dermatologic care are supportive methods for preventing UTI. Also circumcision should be recommended for boys. Third generation cephalosporins are still good options for UTI treatment in patients having no risk factors defined above. The resistance rate is under 20% for ceftriaxone, cefixime, cefoxitin and ESBL positivity rate is 17% in our study group. However, these alarming rates will lead to increasing resistance if antibiotic abuse carries on. Antibiotics should be prescribed for necessary indications with right dose per kilograms and enough duration of treatment. Co- trimoxazole and amoxicillin clavulanate seem to be bad options for UTI management for our population. If there are risk factors as Kızılca et al. defined or antibiotic intake within three months, ESBL positivity must be considered. The patients should be hospitalized if he/she is in bad clinical condition to provide supportive treatment and oral intake. In vivo response of third generation cephalosporines to ESBL (+) *E.coli* may be better than in vitro tests. This situation can be explained by the higher concentrations of antimicrobials in the urine^34^. When there is successful clinical response to the initiated antibiotic (resolution of fever and other symptoms, decreasing trend of acute phase reactants (leukocytes, C- reactive protein) and urine leukocyte count, sterile urine culture at <72 hours), the same agent can be continued even the result of the urine culture is an ESBL (+) agent^35^. Amikacin, gentamicin, carbapenems, piperacillin tazobactam can be ordered for unresponsive and selective cases. Nitrofurantoin, amikacin, gentamicin can be good options for outpatient settings if patient compliance is good. Patients should be monitored for nephrotoxicity and ototoxicity during aminoglycosides therapy. More studies are needed about efficiency of phosphomycin and oral carbapenems in children. Also, phosphomycin susceptibility must be evaluated via agar dilution methods for more accurate results^20^. Good clinical practice and well organized regional surveillance programs are necessary to update the treatment guidelines.

This report has great limitations as it is a retrospective study based on hospital records. The results cannot be generalized to the whole population as only the children visiting our hospital were included in the study. Also, younger children having no toilet training and having defects related to recurrent UTI, the most affected populations from UTI, were excluded. There is lack of data about clinical outcome of the cases. Also, the faecal flora of the cases were not considered although ESBL positivity was reported. We recommend that multicenter surveillance including all pediatric age groups should be conducted in the future with longer clinical follow up.
